# The Recommendation System of Innovation and Entrepreneurship Education Resources in Universities Based on Improved Collaborative Filtering Model

**DOI:** 10.1155/2022/7228833

**Published:** 2022-06-14

**Authors:** Li Geng

**Affiliations:** Xi'an Physical Education University, Xian 710068, China

## Abstract

In the huge number of online university education resources, it is difficult for learners to quickly locate the resources they need, which leads to “information trek.” Traditional information recommendation methods tend to ignore the characteristics of learners, who are the main subjects of education. In order to improve the recommendation accuracy, a recommendation algorithm based on improved collaborative filtering model is proposed in this paper. Firstly, according to the student behavior data, consider the behavior order to create the behavior graph and behavior route. Then, the path of text type is vectorized by the Keras Tokenizer method. Finally, the similarity between multidimensional behavior path vectors is calculated, and path collaborative filtering recommendations are performed for each dimension separately. The MOOC data of a university in China are introduced to experimentally compare the algorithm of the article as well as the control group algorithm. The results show that the proposed algorithm takes better values in evaluation indexes, thus verifying that this algorithm can improve the effectiveness of innovation and entrepreneurship education resources recommendation in universities.

## 1. Introduction

In recent years, the use of online education platform is more and more accepted by learners [1]. Especially, after the outbreak of New Coronavirus, online learning is the preferred learning mode when offline learning cannot be carried out normally. Faced with the huge demand for online learning, online education institutions are providing free online courses and sharing online teaching resources, and the online education industry is showing an explosive growth trend. With the widespread use of online learning platforms, the number of educational resources for innovation and entrepreneurship in universities has also increased dramatically.

In the huge number of online college education resources, learners are difficult to quickly locate the resources they need, resulting in “information trek” [2]. Information recommendation is a method for students to quickly and effectively screen out the objects that meet their preference characteristics from the mass objects. At present, information recommendation technology is widely used in many fields [3]. It is an effective way to solve the problem of “information trek” to apply information recommendation technology to online education and realize personalized recommendation of innovation and entrepreneurship education resources in universities in the process of online education.

With the upgrading of Internet technology, the scale of online education services, represented by Massive Open Online Courses (MOOC), Net Ease Cloud Classroom, and innovation and entrepreneurship education resource platforms built by universities, continues to expand. Meanwhile, teaching data through social media such as Weibo, forum, and post bar also constitute a massive amount of online education resources [4]. These abundant online education service resources not only expand students' knowledge scope and supplement the deficiency of offline learning, but also bring a lot of inconvenience to students. First, complicated online education resources cause “information trek,” and the quality of online education services is uneven, which makes it difficult for students to identify and compare, resulting in poor learning experience and effects. Second, faced with the same high-quality educational resources, students with high information literacy can easily obtain relevant information, while students with low information literacy can hardly access such resources, resulting in new educational inequities in the information environment. Third, all kinds of teaching resources are not pushed to students with the same probability. Mainstream education resources are pushed to students more frequently due to their high frequency of use, thus covering the minority and high-quality education resources. As a result, it is difficult for students to obtain such resources, resulting in “information cocoon.”

How to help learners acquire the knowledge they are interested in quickly and accurately is an urgent problem to be solved by online education service platforms of universities [5]. Educational recommendation services are based on learners' personalized network information and learning behavior. The recommendation algorithm is used to quickly recommend precise college innovation and entrepreneurship education resources that meet students' potential learning needs from the massive educational information resources. It can enhance the experience and effectiveness of online learning [6].

The development of the Internet has broadened the channels for students to obtain information, but not all online information is valuable. Therefore, in the era of information overload, helping students efficiently and accurately screen out the resources they are interested in has become the key to the development of the Internet [7]. Collaborative filtering algorithm is the main technology to solve this problem [8]. As a widely recognized recommendation technology, collaborative filtering can effectively process structured information without studying the content and attributes of the recommendation information. At the same time, it can combine some concepts that are not easy to embody to complete information filtering, and the recommendation has a high degree of intelligence, which fully meets the requirements of personalized recommendation.

Relevant scholars further improve the collaborative filtering recommendation effect by using different methods. A personalized system filtering recommendation method based on coverage minimalism is proposed. According to the method of coverage reduction and student reduction in coverage rough set, the matching of redundant elements and redundant students is realized. Redundant students are removed from the target student set by reduction algorithm to ensure the effectiveness of collaborative filtering. Under the open data set, personalized recommendation services are provided for target students. The literature [9] proposed a method based on the clustering of student interest degree. Combining with the frequency of the target students scoring keywords, the degree of students' preference for keywords is obtained, and the student-keyword preference matrix is constructed, through which the clustering is realized. Logistic function is used to obtain students' interest in the project, determine students' preferences, find out students' similar students in the cluster, and collect some information about neighbours' hobbies to realize the recommendation of target students. However, the above recommendation method has some limitations, such as the serious problem of cold start, and it is difficult to accurately reflect the relationship between students and education resources.

In the era of big data, information overload is a huge problem we are faced with. It is extremely difficult for students to acquire the content they are interested in in time and accurately from massive data [10]. Recommendation system can alleviate the problem of information overload well and has been widely used in e-commerce, online news, and social networking sites.

Personalized recommendation system mainly uses the historical interaction information between students and projects to establish a model to predict students' evaluation of the project. In the traditional recommendation system, the recommendation based on collaborative filtering is the most classic [11]. An intuitive interpretation of collaborative filtering recommendations is that a student's rating of a project can be predicted by the rating of other students who are similar to him. Matrix decomposition is a common collaborative filtering method [12]. The idea of recommendation is to map the student vector and the project vector to a common vector representation space, in which the implicit vector is used to represent the student and the project, and then the score of the project can be directly obtained by calculating the similarity between the implicit vector of the student and the project. The successful application of matrix decomposition model in the recommendation field has led to the emergence of many improved methods based on matrix decomposition in the research field of recommendation system, such as the integration of neighbourhood model in matrix decomposition in the literature [13]. Factorization machine is used for feature interaction to generate more features and add them to model training. The literature [14] improves the effect of matrix decomposition by introducing auxiliary information such as project content. In recent years, the application of deep learning in image, natural language processing, and other fields has achieved good results, and many studies have begun to introduce deep learning into the field of the recommendation system [15]. Deep learning is usually introduced from two perspectives: (1) neural network is used to learn the representation vectors of students and projects, such as the deep matrix factorization (DMF) model proposed in the literature [16]. Dual-path neural network is used to replace the traditional matrix decomposition to learn the representation vectors of students and projects, and then score prediction is made. (2) The neural network is used as the matching function of the recommendation model, and the student vector and the project vector are fed into the matching function to directly obtain the scores of the students on the project, such as the neural collaborative filtering (NCF) model proposed in reference [17]. NeuMF in NCF simply concatenates the student vector and the project vector into a multilayer perceptron (MLP) to calculate the student's rating of the project. The literature [18] comprehensively considered the above two aspects and proposed a deep collaborative filtering model (DeepCF) combining representation learning and function learning. The above collaborative filtering recommendation model and deep learning recommendation model only extract the interaction features between students and projects from a single perspective, and the feature extraction is not comprehensive enough, which will limit the model's recommendation ability.

The essence of recommendation is a continuous decision-making process, and students' interest degree is not constant, so resource recommendation cannot be carried out from a static perspective. Therefore, this paper proposes a recommendation system of innovation and entrepreneurship education resources in universities based on the improved collaborative filtering model.

The innovations and contributions of this paper are listed as follows:

1. According to the student behavior data, consider the behavior order to create the behavior graph and behavior route.
2. Use Keras Tokenizer to vectorize the path of text type.
3. The similarity between multidimensional behavior path vectors is calculated, and path collaborative filtering recommendations are performed for each dimension separately.

This paper consists of five main parts: the first part is the introduction, the second part is state of the art, the third part is methodology, the fourth part is result analysis and discussion, and the fifth part is the conclusion.

## 2. State of the Art

In order to improve the expansibility of the recommendation system, a scholar proposed Spark Hierarchical CF, which uses the student preference model and clustering algorithm to divide students into different student clusters according to different preference characteristics. And then it makes collaborative filtering recommendations for different student clusters. Compared with the algorithm based on MapReduce, the recommendation accuracy is improved and the operation time is saved, but the centre point of clustering is difficult to determine.

At present, there are four kinds of research on student behavior path, including methods based on reinforcement learning, methods based on statistical analysis, methods based on activity trajectory clustering, and methods based on the ant colony algorithm.

In order to improve the problem that the collaborative filtering algorithm ignores item attributes, a dynamic individual recommendation method based on reinforcement learning was proposed in the literature [19]. Firstly, students' attribute labels are mined from the operation behaviors, and then the reward and punishment models of attribute labels are adjusted according to the operate path and recall path to achieve dynamic adjustment of label weight. Finally, reinforcement learning is used to achieve label recommendation. The disadvantage of this method is that only recent behaviors are considered. Student preference information implied by behavioural pathways is not fully utilized.

The literature [20] proposed that students' experience perception can improve the recommendation effect. According to the behavioural data, analyse the student behavior path and create the student experience perception model. The behavioural path is defined as continuous indicators such as attracting attention, expressing interest, clicking intention, and watching video, which can better show the development trend of students' interest. However, this method has the problems of cold start and sparse data.

Considering the similarity between student visit and ant foraging, the ant colony algorithm was introduced in the literature [21] to realize recommendation, and the transition probability was calculated according to students' browsing behavior and preferred browsing path, so as to dynamically make recommendation. However, there are cold start problem and Matthew effect in this method, and there is occasional error in recommendation based on transfer probability. The literature [22] imitates the concept of pheromone in the ant colony algorithm and proposes pheromone-based algorithm to combine students' browsing behavior with reading behavior through commodity pheromone, and then recommend students according to their browsing trajectory. However, the properties of resource pheromones lead to the Matthew effect.

## 3. Methodology

**Figure 1:**
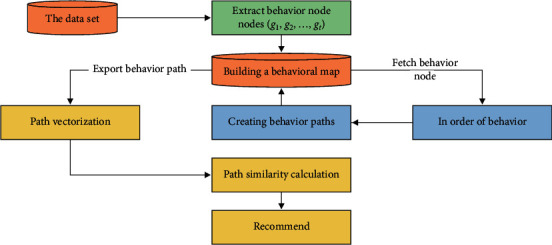
Flowchart of the BR-CF algorithm.

This paper proposes a behavior path collaborative filtering recommendation algorithm based on knowledge graph. First, *t* behaviors in user behavior data are initialized to *t* nodes = *g*_1_, *g*_2_,…, *g*_*t*_, each node *g* stores the attribute of this behavior. And these nodes are constructed into a behavior knowledge map in Neo4j, which is referred to as the behavior map. Second, define the behavior path for the user against the same target entity in long weeks Route_uid,iid_=(*h*_1_, *h*_2_,…, *h*_*t*_), and construct it according to the behavior sequence in the behavior graph. Uid and iid indicate the student ID and education resource ID, respectively. Then, the behavior path is derived for vectorization. Finally, the similarity between multidimensional behavior paths is calculated. The flow of this algorithm is shown in Figure 1. This section describes the specific steps of the algorithm.

### 3.1. Construct Behavior Map

**Figure 2:**
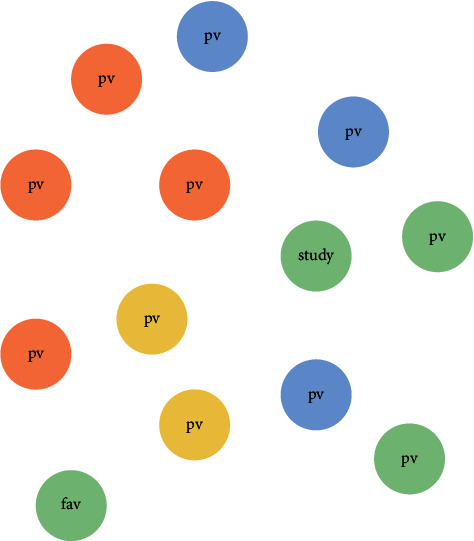
Preliminarily constructed behavior map.

Taking each behavioural datum as a node, *w* genera of each behavior (*f*_1_, *f*_2_,…, *f*_*w*_) is sealed as nodes and stored in the Neo4j data library. The construction algorithm is shown in

(1) $$\begin{array}{c} g=\text{Graph}\left(\text{host,http\_port,user,password}\right),\\ \text{node}=\text{Node}\left({}^{\prime }f1{}^{\prime }=\;f1,{}^{\prime }f2{}^{\prime }=f2,\ldots ,{}^{\prime }fm{}^{\prime }=fm\right),g.\text{create}\left(\text{node}\right),\\ g.\text{create}\left(\text{node}\right), \end{array}$$

where host, http_ port, user, and password represent host address, port address, database user name, and password, respectively, which are used to connect to the neo4j database. Node encapsulates each behavior datum into a node, and the create (node) command stores each node in the neo4j database. The preliminarily constructed behavior map is shown in Figure 2.

### 3.2. Creating a Behavior Path

First, prioritize your actions. According to *t* behavior types contained in the behavior map, the behavior priority is defined (*h*_1_, *h*_2_, *h*_3_,…, *h*_*t*_), *h* represents a single behavior, *h*_1_ has the lowest priority, and *h*_*t*_ has the highest priority.

Then, define the behavior path. This paper defines the behavior path as an ordered set of all behaviors generated by students for the same educational resource in a long period. Route_uid,id_=(*h*_1_, *h*_2_,…, *h*_*t*_). Uid and iid represent the student ID and education resource ID, respectively.

**Figure 3:**
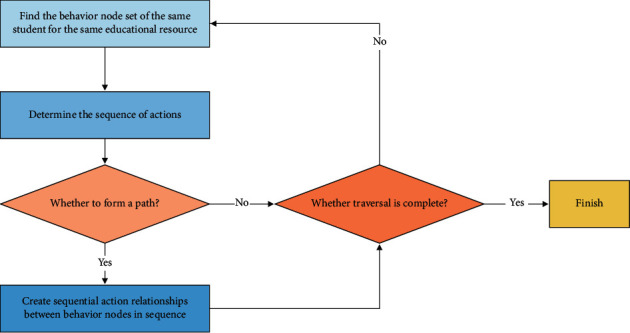
Steps to create a behavior path.

**Figure 4:**
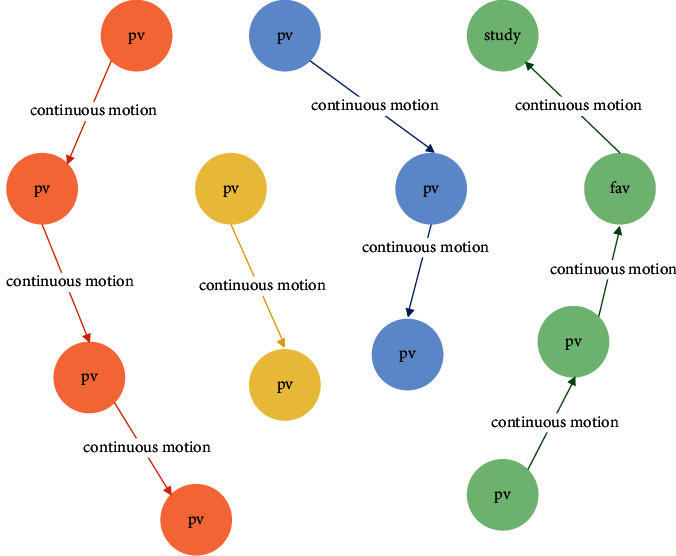
Behavior path diagram.

Finally, create a behavior path. According to the behavior sequence, traverse the behavior map, create a “continuous action” relationship for the continuous behavior made by the same student about the same educational resources, skip the single node that does not constitute a path, and get the Route_uid, iid_ of all students about different educational resources after completing the traversal. The specific steps are shown in Figure 3. The created behavior path is shown in Figure 4.

### 3.3. Path Vectorization

Because the data type of the behavior path created in the previous step is text, and the similarity calculation requires digital data. The next is the vectorization and alignment of paths.

Firstly, classify the behavior paths by behavior priority, as listed in Table 1.

Secondly, vectorize each path in each class from text format to digital format. In this paper, Keras's Tokenizer class is used for vector transformation.

Finally, vector alignment is required. Because the path length is different, the length of each vector is also different, which means that the spatial dimension of the vector is not unified, and the similarity cannot be calculated. Calculate the maximum path length of all behavior paths and use Keras's kps.pad_sequences method to make the spatial dimension of each path the same as the maximum length, with the principle of complement 0. The data change process is listed in Table 2.

### 3.4. Calculation of Path Similarity

Here, the path similarity is measured by the sum of the distances between a path in class *G* and each path in class *H*. As shown in equation (3), the smaller the total distance, the greater the similarity.

As shown in Table 2, the array of class *h*_2_ and class *h*_3_ is empty, it can be seen that there may be empty classes in the path classification. Direct calculation of similarity will lead to empty objects. Therefore, it is necessary to distinguish which classes are empty in the path classification and finally decide which behaviors can be recommended.

According to the permutation and combination principle, there are 2^*T*^ − 1 types of classification for *T* types of behavior. For example, there are 15 types of classification for 4 types, as listed in Table 3, where combination (*h*_1_) means that all classes except fot *h*_1_ are empty, and combination (*h*_2_, *h*_3_, *h*_4_) means that *h*_2_, *h*_3_, and *h*_4_ are not empty. The *h*_1_ class is empty, and so on.

After the path combination is determined, the path similarity is calculated. In this paper, Euclidean distance is used to calculate the similarity of paths. The calculation formula of Euclidean distance in *t*-dimensional space is shown in

(2) $$\begin{array}{c} d\left(i,j\right)=\sqrt{\sum_{x=1}^{t}{\left(i_{x}-j_{x}\right)}^{2}}. \end{array}$$

Secondly, the sum of the distance between each path in class_1_ and the path in class_2_ is calculated, as shown in (3), where class_1_ and class_2_ represent two classes in the path subclass. The classification of other paths is similar:

(3) $$\begin{array}{c} \text{distances}=\sum_{i=1}^{t}d\left(i,j_{x}\right),\;i\in {\text{class}}_{1},\;j\in {\text{class}}_{2}. \end{array}$$

Finally, the corresponding total distance list can be obtained for *h*_t_ class path, as shown in

(4) $$\begin{array}{c} \text{List\_distances}\_h_{t}=\left[{\text{distances}}_{1},{\text{distances}}_{2},\ldots \right],\;t\in \left[1,t\right]. \end{array}$$

If the combination is (*h*_2_, *h*_3_, *h*_4_), the total distance list can be obtained by calculation, as shown in

(5) $$\begin{array}{c} \text{List\_distances}\_\;h_{2}=\left[{\text{distances}}_{1},{\text{distances}}_{2},\ldots ,{\text{distances}}_{w}\right],\\ \text{List\_distances}\_\;h_{3}=\left[{\text{distances}}_{1},{\text{distances}}_{2},\ldots ,{\text{distances}}_{t}\right], \end{array}$$

where *w* and *t* are the number of paths in *h*_2_ and *h*_3_, respectively. List_distances_*h*_2_ represents the total distance list between each path in *h*_2_ and all paths in *h*_3_. And List_distances_*h*_3_ represents the total distance list between each path in *h*_3_ and all paths in *h*_4_.

### 3.5. Multidimensional Recommendation

In this paper, the definition is derived from the multidimension (*d*_1_, *d*_2_, *d*_3_,…, *d*_*t*−1_). *D* represents a set of recommendations that may be generated in line *h*. The *d*_1_ pair should be able to generate the *h*_2_ recommendation set. *D*_*t* −1_ corresponds to the possible recommendation set of *h*_*t*_, which can be divided into possible_*h*_*t*_ table. *D* is the set of target entities corresponding to min (List_distances), that is, the set of target entities corresponding to the path with the minimum total distance. The results of recommendation obtained are shown in

(6) $$\begin{array}{c} \left\{d_{1},d_{2},\ldots ,d_{n-1}\right\}=\left\{\text{possible}\_h_{2}{}^{\prime \prime }:\left[\cdots \cdots \right],\right)\\ {}^{\prime \prime }\text{possible}\_h_{3}{}^{\prime \prime }:\left[\cdots \cdots \right],\\ \cdots \cdots \\ \left({}^{\prime \prime }\text{possible}\_h_{t}{}^{\prime \prime }:\left[\cdots \cdots \right]\right\}. \end{array}$$

According to the example results in equation (5), there are two recommendation dimensions *d*_2_ and *d*_3_. The returned recommendation results are shown in equation (7), which, respectively, represents the set of educational resources where the user recommended by this algorithm may have behaviors *h*_3_ and *h*_4_, and the value is the ID or other unique identification of the educational resource:

(7) $$\begin{array}{c} \left\{d_{3},d_{4}\right\}=\left\{{}^{\prime \prime }\text{possible}\_h_{3}{}^{\prime \prime }\left[\begin{array}{c} 2733371,332733 \end{array}\right]\right),\\ \left({}^{\prime \prime }\text{possible}\_h_{4}{}^{\prime \prime }:\left[124451\right]\right\}. \end{array}$$

The item collaborative filtering recommendation algorithm, referred to as item CF, is used to recommend educational resources similar to those they liked before to college students.

In order to further analyse the recommendation performance of BR-CF, item CF and BR-CF are cascaded and mixed, respectively, in this section, and an improved algorithm based on the combination of item CF and BR-CF is proposed.

**Figure 5:**
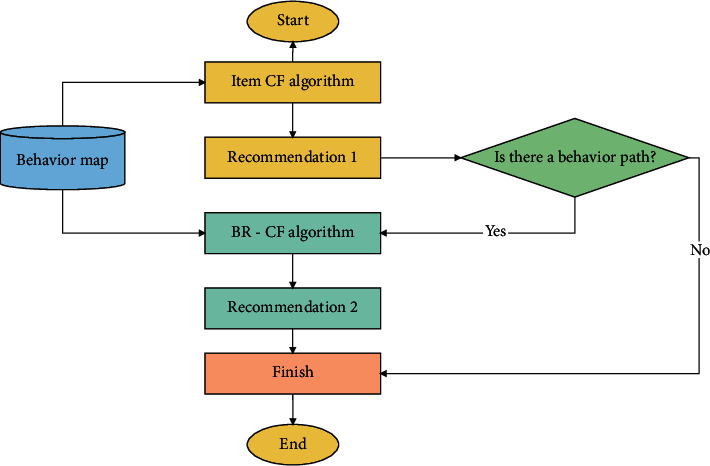
Flowchart of combine the item CF and BR-CF algorithm.

The cascade recommendation algorithm of item CF and BR-CF is referred to as Combine item CF and BR-CF. In the recommendation result of resource collaboration, the algorithm further calculates the set of target entities with behavior paths and the minimum total distance from the behavior paths of all target entities in the verification set. The flowchart is shown in Figure 5.

## 4. Result Analysis and Discussion

The experimental data in this paper are from a MOOC of a university in China bound by QQ account, and the crawler tool is used to directly obtain student information and innovation and entrepreneurship education resource data of universities. A total of 36,967 pieces of online learner information were obtained, including major, educational background, geographical location, friend relationship, learning time, course name, and evaluation content. After data screening, the dormant accounts and abandoned accounts whose monthly login times are less than 3 are excluded. Finally, the effective data set obtained contains 2400 students. The students were divided into two data sets in the ratio of 8 : 2. Among them, 1920 students' information constituted the training data set, and the other 480 students' information constituted the test data set.

In order to test the quality of the proposed algorithm, the proposed algorithm based on the improved collaborative filtering model is compared with the literature [23], literature [24], and literature [25]. The number of adjacent recommendation students is set as 5, and the value is increased by 15 until it is 50. The Precision, Recall, and RMSE values of the four recommendation models under different number of recommendation students are listed, as shown in Table 4.

**Figure 6:**
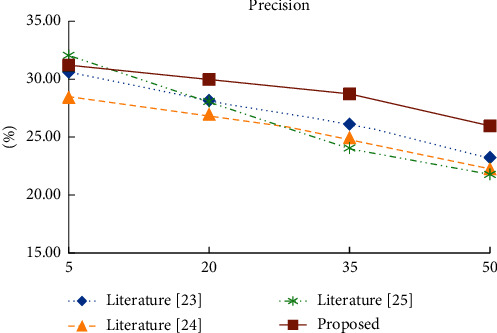
Accuracy of four recommendation algorithms under different recommendation numbers.

**Figure 7:**
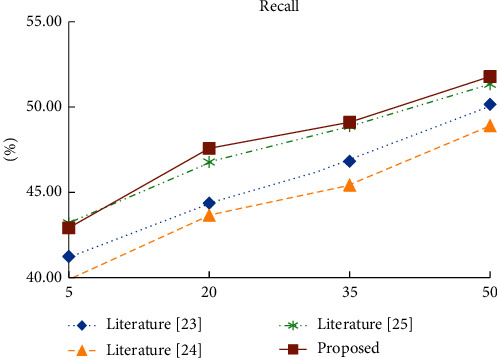
Recall rate of four recommendation algorithms under different recommendation numbers.

**Figure 8:**
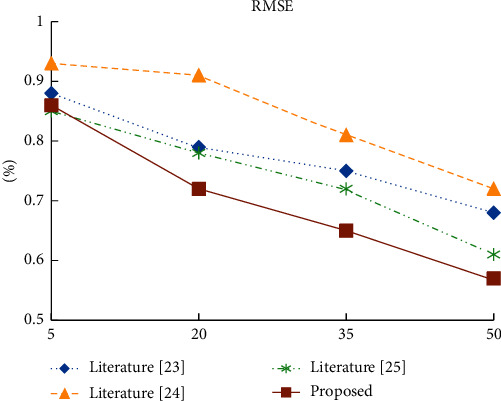
Root mean square error of four recommendation algorithms under different recommendation numbers.

As shown in Figures 6–8, when the number of recommended students is greater than 20, the accuracy and root mean square error of the proposed algorithm in experimental data are better than those of the other three recommendation algorithms. In terms of recall rate, when the number of recommended students is greater than 35, there is not much difference between the proposed algorithm and the knowledge-based recommendation algorithm, but it is significantly better than the other two algorithms. Compared with the currently commonly used recommendation algorithm, the proposed algorithm takes into account student information, social relationship, and online learning behavior, so the recommendation quality is higher.

## 5. Conclusion

At present, there are a lot of learning resources in online education learning platform, but the personalized service is not high. The target of online education services is learners. The commonly used personalized recommendation algorithm of education services only considers static information or dynamic characteristics of users, resulting in low recommendation quality. The collaborative filtering algorithm is the mainstream of information recommendation technology at present. However, the traditional recommendation system based on collaborative filtering algorithm has some limitations, such as low accuracy and cold start. And it is difficult to accurately reflect the relationship between students and educational resources. In order to improve the recommendation accuracy, this paper proposes a recommendation algorithm based on the improved collaborative filtering model for innovation and entrepreneurship education resources in universities. This paper uses 20,059 real behavior data and successfully creates 2,266 behavior paths according to the sequence of students' behaviors, which enriches the semantic information of the data. In addition, students' behavior data are fully used to make recommendations from three dimensions, which improves the data utilization rate and the diversity of recommendations. In particular, this paper puts forward the concept of behavior path, which can well represent the evolution and development process of students' behavior and better predict students' learning preferences by creating associations between behaviors. In order to test the quality of the recommendation algorithm, the proposed algorithm and other three recommendation algorithms were used as the control group in the experiment, and the Precision, Recall, and RMSE of these four recommendation algorithms were calculated under different recommended users. It shows the recommendation the proposed algorithm has more advantages in three evaluation indicators, and can be applied to online education services to better provide students with high-quality recommendation of college innovation and entrepreneurship education resources. Since the experimental platform and data in this paper are relatively single, in order to further verify and improve the personalized recommendation effect of the algorithm in this paper, other online education service platforms will be introduced in the future to verify the scalability and accuracy of this method.

## Figures and Tables

**Table 1:** Path classification.

Classification	Classification basis
Class *h*_1_	The highest behavior priority in the path is *h*_1_
Class *h*_2_	The highest behavior priority in the path is *h*_2_
…	…
Class *h*_*t*_	The highest behavior priority in the path is *h*_*t*_

**Table 2:** Example of data change process.

Path status	Numerical value
Initial behavior path set	{“*h*_1_–*h*_2_–*h*_4_–*h*_1_–*h*_1_” “*h*_1_–*h*_1_–*h*_1_”}
	{*h*_1_:[“*h*_1_–*h*_1_–*h*_1_”]

After path classification	*h*_2_:,
	*h*_3_:,
	*h*_4_:[“*h*_1_–*h*_2_–*h*_4_–*h*_1_–*h*_1_”]}
	{*h*_1_:[[1, 1, 1]]

After path steering	*h*_2_:,
	*h*_3_:,
	*h*_4_:[[1, 2, 4, 1, 1]]}
	{*h*_1_:[[1, 1, 1, 0, 0]],

After path alignment	*h*_2_:,
	*h*_3_:,
	*h*_4_:[[1, 2, 4, 1, 1]]}

**Table 3:** Path category combination.

Possible combination of route categories	
1	(*h*_1_)
2	(*h*_2_)
3	(*h*_3_)
4	(*h*_4_)
5	(*h*_1_, *h*_2_)
6	(*h*_1_, *h*_3_)
7	(*h*_1_, *h*_4_)
8	(*h*_2_, *h*_3_)
9	(*h*_2_, *h*_4_)
10	(*h*_3_, *h*_4_)
11	(*h*_1_, *h*_2_, *h*_3_)
12	(*h*_1_, *h*_2_, *h*_4_)
13	(*h*_1_, *h*_3_, *h*_4_)
14	(*h*_2_, *h*_3_, *h*_4_)
15	(*h*_1_, *h*_2_, *h*_3_, *h*_4_)

**Table 4:** Comparison of experimental results of four recommended algorithms with different TOP-N.

TOP-N	Index	Literature [23]	Literature [24]	Literature [25]	Proposed
5	Precision (%)	30.61	28.42	32.05	31.21
	Recall (%)	41.24	39.94	43.21	42.92
	RMSE	0.88	0.93	0.85	0.86

20	Precision (%)	28.16	26.96	28.08	29.98
	Recall (%)	44.37	43.65	46.78	47.58
	RMSE	0.79	0.91	0.78	0.72

35	Precision (%)	26.12	24.94	24.06	28.73
	Recall (%)	46.82	45.42	48.88	49.11
	RMSE	0.75	0.81	0.72	0.65

50	Precision (%)	23.24	22.25	21.78	25.97
	Recall (%)	50.16	48.9	51.34	51.79
	RMSE	0.68	0.72	0.61	0.57

## Data Availability

The labeled dataset used to support the findings of this study are available from the corresponding author upon request.
